# Observational Learning in Low-Functioning Children With Autism Spectrum Disorders: A Behavioral and Neuroimaging Study

**DOI:** 10.3389/fpsyg.2018.02737

**Published:** 2019-01-09

**Authors:** Francesca Foti, Fabrizio Piras, Stefano Vicari, Laura Mandolesi, Laura Petrosini, Deny Menghini

**Affiliations:** ^1^Department of Medical and Surgical Sciences, Magna Græcia University of Catanzaro, Catanzaro, Italy; ^2^IRCCS Fondazione Santa Lucia, Rome, Italy; ^3^Child Neuropsychiatry Unit, Neuroscience Department, Children’s Hospital Bambino Gesù, Rome, Italy; ^4^Department of Motor Sciences and Wellness, Università degli Studi di Napoli Parthenope, Naples, Italy; ^5^Department of Psychology, Sapienza University of Rome, Rome, Italy

**Keywords:** imitation, social learning, learning, hyperimitation, mirror neuron system, cerebellum

## Abstract

New skills may be learned from the outcomes of their own internally generated actions (experiential learning) or from the observation of the consequences of externally generated actions (observational learning). Observational learning requires the coordination of cognitive functions and the processing of social information. Due to the “social” abilities underlying observational learning, the study of this process in individuals with limited social abilities such as those affected by Autism Spectrum Disorders (ASD) is worthy of being investigated. We asked a group of 16 low-functioning young children with ASD and group of 16 sex- and mental age-matched typically developing (TD) children to build a house with a set of bricks after a video-demonstration showing an actor who built the house (observational task – OBS task) and then to build by trial and error another house (experiential task – EXP task). For ASD group, performances in learning tasks were correlated with measures of cortical thickness of specific Regions of Interest (ROI) and volume of deep gray matter structures known to be related with such kinds of learning. According to our *a priori* hypothesis, for OBS task we selected the following ROI: frontal lobe (pars opercularis, pars triangularis, and premotor area), parietal lobe (inferior parietal gyrus), temporal lobe (superior temporal gyrus), cerebellar hemispheres. For EXP task, we selected the following ROI: precentral frontal gyrus and superior frontal gyrus, cerebellar hemispheres, basal ganglia, thalamus. Although performances of ASD and TD children improved in both OBS and EXP tasks, children with ASD obtained lower scores of goal achievement than TD children in both learning tasks. Only in ASD group, goal achievement scores positively correlated with hyperimitations indicating that children with ASD tended to have a “copy-all” approach that facilitated the goal achievement. Moreover, the marked hyperimitative tendencies of children with ASD were positively associated with the thickness of left pars opercularis, left premotor area, and right superior temporal gyrus, areas belonging to mirror neuron system, and with the volume of both cerebellar hemispheres. These findings suggest that in children with ASD the hyperimitation can represent a learning strategy that might be related to the mirror neuron system.

## Introduction

New skills may be learned from the outcomes of their own internally generated actions (“experiential learning” or “learning by doing” or “learning by trial and error”) or from the observation of the consequences of externally generated actions (“observational learning”) (Bandura, 1977; Petrosini, 2007; Meltzoff et al., 2009). The main difference between mechanisms is that action and its consequences are experienced by the learners themselves in the case of experiential learning, or by another individual in the case of observational learning (Buchanan and Dean, 2010).

Observational learning requires the coordination of complex cognitive functions (as action representation, attention, motivation) and the processing of social information (as understanding others’ gestures and making inferences about their behaviors) (Gallese and Goldman, 1998; Meltzoff and Decety, 2003). Due to the social abilities underlying observational learning, the study of this process in individuals with limited social abilities such as those affected by Autism Spectrum Disorders (ASD) is worthy of being investigated. In fact, despite the heterogeneity in clinical presentation, one unifying feature of ASD conditions is the abnormality of social interaction including imitation, joint attention, goal understanding, affect sharing, communicative use of language and play (Rogers and Williams, 2006; Mundy, 2011; Nadel et al., 2011; Vivanti et al., 2014, 2016). Many individuals affected by ASD do not attend to environmental stimuli at a level sufficient to learn a range of (pro-)social behaviors through observation of others (Vivanti and Dissanayake, 2014). Several studies showed that individuals with ASD show less or impaired imitation in a variety of tasks (Williams et al., 2004; Nadel, 2015), although other reports indicated increased imitation in ASD, with behaviors (echopraxia) and speech (echolalia) imitated from others, without regarding the context and meaning of the actions (Williams et al., 2004; Bellini and Akullian, 2007; Ledford et al., 2008; Taylor and DeQuinzio, 2012; Plavnick and Hume, 2014). Recently, we analyzed the features of learning by observation and learning by doing in high-functioning children with ASD who learned a visuo-motor sequence after observing an actor who detected it by trial and error (observational training) or directly by doing (Foti et al., 2014). Children with ASD were impaired in learning by doing the task, whereas they were as efficient as typically developing children in learning by observation the task. Specifically, through the observational training, children with ASD learned to put into action the correct decision making and the appropriate strategies to discover rules and generate new knowledge to be automated. In spite of the beneficial effects of the observational training, the children with ASD reproduced also patently wrong actor’s actions, showing thus marked tendencies to hyperimitate (Foti et al., 2014).

Whereas research has focused on brain structures and mechanisms of experiential learning, relatively less is known regarding how observational learning proceeds. In particular, several neuroimaging studies have established that learning by trial and error engages cortical and subcortical structures organized into two main circuits where dynamic changes occur during motor learning/adaptation: the cortico-striato-thalamo-cortical loop and the cortico-cerebello-thalamo-cortical loop (Doyon et al., 2003). In parallel, the prefrontal cortex is particularly involved in the storage and retrieval from long-term memory of known rules for action (Bunge et al., 2003; Bunge, 2004; Donohue et al., 2005; Crone et al., 2006). Conversely, observational learning is thought to utilize brain regions responsive to both observation and execution of action (potential mirror neuron system, MNS; Rizzolatti et al., 1996, 2001; Rizzolatti and Craighero, 2004). MNS includes premotor cortex (PMC), inferior frontal gyrus (IFG), and inferior parietal lobule (IPL), areas which receive their main visual input from the superior temporal sulcus (STS) (Brass and Heyes, 2005; Caspers et al., 2010; Molenberghs et al., 2010). To this core network of brain regions with mirroring properties, further areas, as the cerebellum, have been added (Iacoboni and Dapretto, 2006). Insofar as the MNS generates a simulation circuit that allows the association between one’s own actions with others’ actions, MNS is retained to be involved in action understanding and imitation, social interaction, identification of others’ emotions and language comprehension (Iacoboni, 2005, 2009; Rizzolatti and Sinigaglia, 2010).

Over the past few years, it has been hypothesized that the social ‘aloneness’ and the imitative impairment of individuals with ASD might result from abnormalities in frontal and temporal regions – commonly referred to as “social brain” – as well as in MNS (Oberman et al., 2005, 2008; Dapretto et al., 2006; Hamilton et al., 2007; Leighton et al., 2008; Fan et al., 2010; Hamilton, 2015). However, the studies have produced controversial findings probably because of the structural heterogeneity within the syndrome, the different ages of the cohorts examined, the neuroimaging techniques used, the largely lacking correlations between neuroanatomical abnormalities and behavioral findings. Furthermore, gathering high-quality neuroimaging data from low-functioning young children with ASD has proven to be a demanding challenge (Schumann et al., 2010; Hazlett et al., 2011; Calderoni et al., 2012).

**FIGURE 1:**
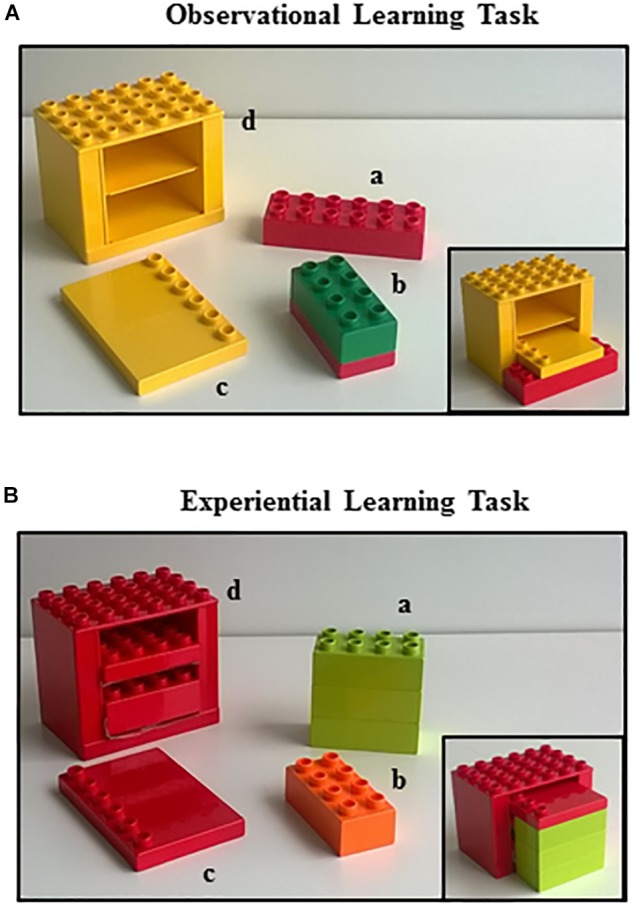
Learning tasks. The Lego^®^ Duplo bricks used in the observational learning task **(A)** and in the experiential learning task **(B)**. In the lower right corner of each figure is represented the final house.

**FIGURE 2:**
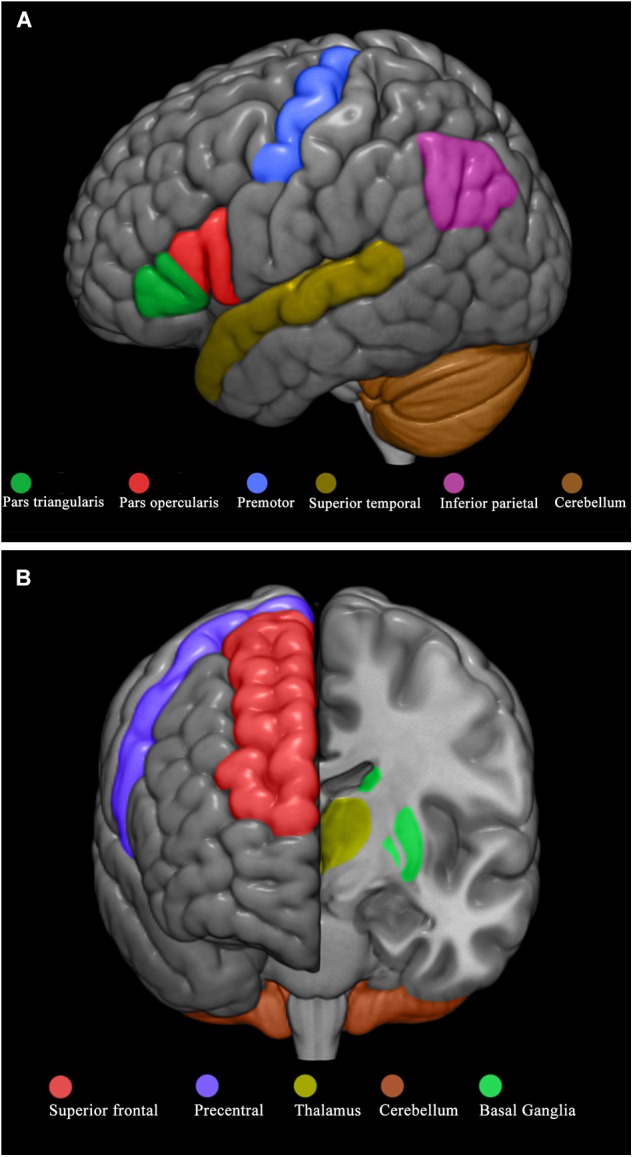
Brain regions selected for the observational and experiential learning tasks. Brain regions selected for the observational learning task **(A)** and experiential learning task **(B)**.

Starting from this evidence, the present study aimed at addressing the issue of the observational learning and experiential learning in the presence of ASD by means of a behavioral and neuroimaging approach. Specifically, we asked a group of 16 low-functioning young children with ASD and a group of 16 sex- and mental age-matched typically developing (TD) children to build a house with a set of bricks after a video-demonstration showing an actor who built by trial and error a house with the same set of bricks (Figure 1A). Subsequently to assess the experiential learning, the children were asked to build by trial and error another house with different bricks (Figure 1B). Then, only for ASD group, to correlate behavioral performances with morphological brain measures, behavioral outcomes in both learning tasks were correlated with measures of cortical thickness of specific Regions of Interest (ROI) and volume of deep gray matter structures known by the literature (*a priori* hypothesis) to be related with learning tasks. According to our *a priori* hypothesis, for the observational learning task we selected the following ROI: frontal lobe (pars opercularis, pars triangularis, and premotor area), parietal lobe (inferior parietal gyrus), temporal lobe (superior temporal gyrus), and cerebellar hemispheres (Figure 2A). For the experiential learning task, we selected the following ROI: precentral frontal gyrus and superior frontal gyrus, cerebellar hemispheres, basal ganglia (caudate, pallidum, and putamen), and thalamus (Figure 2B). Notably, the association of behavioral and neuroimaging data obtained from the very same young children is a paradigm scarcely present in literature.

## Materials and Methods

### Participants

Sixteen children with ASD (14 males) with a mean chronological age (CA) of about 7 years (88.2 months ± SD 33.3), mental age (MA) of 48.4 months ± 15.4, and IQ of 67 ± 21.7 and 16 TD children matching the ASD children for MA (47.2 ± 14.7) and sex (14 males) participated in the present study (Table 1). In the TD children, mean IQ was 103.4 ± 7.9 and mean CA was 49.3 ± 13.9. Children with ASD were recruited at the Children’s Hospital Bambino Gesù in Rome, while the TD children were recruited in a kindergarten in Rome. To determine the children’s cognitive development level the Griffith Mental Developmental Scale for ages 2–8 (GMDS-ER 2–8, Luiz et al., 2006) was used. All participants were diagnosed according to the DSM-IV TR diagnostic criteria (American Psychiatric Association [APA], 2000). Module 1 of Autism Diagnostic Observation Schedule (ADOS, Lord et al., 2005) was used to confirm the diagnosis of autistic spectrum disorder (ASD, scores 7–10) or Autistic disorder (A, scores >10). Based on the ADOS results, 11 children met the criteria for A and 5 for ASD (Table 1). In the current study, all 16 children are named ASD. The ADOS was administered and scored by licensed clinicians that had reached clinical reliability on the instrument. Adaptive behavior was assessed using the survey form of the Vineland Adaptive Behavior Scales (VABS, Sparrow et al., 1994). All participants had normal or corrected-to-normal vision and were screened for exclusion criteria (epilepsy, and any other neurological or psychiatric condition) prior to taking part of the research.

**Table 1:** Description of ASD children.

					ADOS				VABS (in months)			
	Gender of participants	CA (months)	MA (months)	IQ	Total	Communication	Social interaction	Stereotypies	Communication	Daily living	Socialization	Motor
A	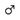	94	62	67	13	3	7	1	28	33	22	51
	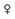	65	57	70	18	6	12	3	48	37	42	51
	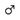	59	37	75	16	4	12	2	29	33	39	49
	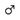	132	-	50	17	5	12	5	20	38	20	39
	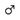	130	69	71	14	4	10	3	34	27	32	36
	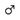	96	58	63	20	9	11	6	31	30	26	49
	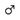	86	28	39	20	6	14	6	23	29	23	36
	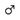	87	55	74	13	2	11	3	37	56	31	63
	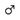	136	-	50	18	4	14	6	18	28	20	31
	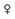	155	62	44	20	7	13	5	48	37	21	47
	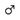	73	25	42	15	5	10	2	34	27	32	36
ASD	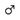	74	36	58	9	2	7	2	18	32	21	37
	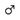	51	27	61	8	2	6	5	21	25	24	25
	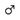	48	38	68	10	3	7	1	24	20	28	33
	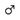	67	55	74	10	3	7	5	27	38	20	43
	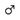	59	41	71	10	3	8	2	28	31	24	52
	**Mean ± SD**	88.25 ± 33.31	46.43 ± 14.84	58.85 ± 12.68	14.50 ± 4.12	4.25 ± 1.98	10.06 ± 2.72	3.56 ± 1.82	29.25 ± 9.28	32.56 ± 8.03	26.56 ± 6.89	42.38 ± 9.86


*ASD, autistic spectrum disorder; A, autistic disorder; CA, chronological age; IQ, intelligence quotient; MA, mental age; ADOS, Autism Diagnostic Observation Schedule; VABS, Vineland Adaptive Behavior Scales. All participants had normal or corrected-to-normal vision and were screened for exclusion criteria (epilepsy, and any other neurological or psychiatric condition) prior to taking part of the research.*

The study was approved by the Ethics Committee of Children’s Hospital “Bambino Gesù,” Rome, Italy [protocol number: 486LB] and was conducted according to the Declaration of Helsinki. Parents of the participants gave informed written consent.

### Learning Tasks and Experimental Procedures

All children readily participated in the study and willingly performed the experimental tasks. The tasks consisted in building a little house with Lego^®^ Duplo bricks provided to children by the experimenter. In the observational task, the participants learned to build a house by assembling the bricks after observational training in which they observed a video showing an actor (FF) who built the house (Observational Learning – OBS task). In the experiential task, the participants assembled a different set of Lego Duplo bricks to build by trial and error a different house without any observational training (Experiential Learning – EXP task).

#### Task Materials

In the OBS task, four colorful interlocking plastic Lego^®^ Duplo bricks were presented on a table: (a) a red brick (2 × 6) locking connector; (b) a green and red brick (2 × 4) formed by two bricks already assembled one above another; (c) a yellow flat tile (1 × 6); and (d) a yellow frame (4 × 6) representing the main structure of the house (Figure 1A). In the EXP task, a different set of four colorful interlocking plastic Lego^®^ Duplo bricks were presented on the table: (a) a green brick (2 × 4) formed by three bricks already assembled one above another; (b) an orange brick (2 × 4); (c) a red flat tile (1 × 6); and (d) a red frame (4 × 6) representing the main structure of the house (Figure 1B). Notably, in both tasks the piece “b” was superfluous and it had not to be used in building the house.

#### Task Procedures

The participant sat in front of a table in a lighted quiet room at the Neuropsychiatric Unit of the Bambino Gesù Children’s Hospital of Rome, Italy. The TD children were individually tested in a quiet room at their school. For the OBS task, the Lego^®^ bricks (Figure 1A) were first provided to the participant for about 30 sec to allow familiarization with the experimental material. Afterward, the experimenter (FF) removed the bricks and put a computer in front of the child at a distance of 60 cm to show a brief video (1 min) in which an actor (FF) built the house by trials and errors by interlocking the bricks the child had seen and familiarized with immediately before. The video of the observational training showed the actor who built the house making some evidently wrong actions (errors) as trying to insert a big piece into a small one or trying to insert wrongly interlocked bricks beating them against the house main structure. Remarkably, the actor made some actions superfluous for building the house: namely, she showed off the single bricks displaying them one-by-one, she emphatically performed head movements of assent or denial that emphasized the correctness or the incorrectness of her actions. After the observational training, the participant received again the bricks and the child was required to build the house. There was no fixed time limit for executing the task. The entire OBS task included three trials (in each trial children saw the same video) with an inter-trial interval of about 5 min.

At the end of the OBS task, the child could leave the room for about 15 min. Afterward, the participant performed the EXP task in which he/she had to build a different house with a new set of bricks (Figure 1B). After the familiarization phase with the new bricks (30 s), the experimenter removed the bricks and a single still image representing the completely built house appeared on the computer screen and remained for 20 s. Then, the participant received again the bricks to build the second house. The entire EXP task included three trials with an inter-trial interval of about 5 min.

Children’s behavior was video-recorded during both tasks. This has permitted to score the children’s performance in both task and to monitor the children’s attention to the video during the observational training in the OBS task and to the single still image in the EXP task. The choice for using video had different reasons. Firstly, numerous studies have shown that video-demonstrations are more effective than live-demonstrations in teaching various tasks to children with ASD, in contrast to typically developing children that learn faster and better with live-demonstrations (Charlop-Christy et al., 2000; Hayne et al., 2003; Ayres and Langone, 2005; Klein et al., 2006; Bellini and Akullian, 2007; Delano, 2007; McCoy and Hermansen, 2007). Secondly, because all participants receive exactly the same information (same gestures performed by the same actor at the same speed), video-demonstrations are more rigorous and stringent than live-demonstrations. Thirdly, given the bricks were not available during the video-demonstration, it prevented the child from trying to get the bricks during the observational training.

### Behavioral Parameters

In each trial of OBS and EXP tasks we measured: *goal achievement*, assigning a score ranging from 1 (worst value) to 3 (best value) to the child’s performance. In particular, we assigned the score: 1. when only goal-irrelevant *actions* (neither interlocking bricks nor inserting them in the main house structure) were performed; 2. when the child wrongly assembled bricks or attempted to insert wrong bricks in the main house structure; and 3. when the child succeeded in inserting the correctly assembled bricks in the main house structure.

In each trial of the OBS task, we measured *hyperimitations*, that is, the number of wrong or useless actor’s actions which the child mimicked (i.e., the child could show off the bricks, move his/her head performing movements of assent or denial, beat the wrong bricks against the house main structure, or try to insert a big piece into a small one, as previously observed in the actor’s performance). Summing the hyperimitations of the three trials, we obtained the *total hyperimitations* to be correlated with neuroanatomical values.

Moreover, in each trial of the OBS task, we measured the *imitated sequences.* To this aim we first segmented the chain of actions performed by the actor (1: showing off the bricks; 2: assembling wrong bricks; 3: beating the wrong bricks against the house main structure; 4: moving the head performing movements of denial; 5: assembling right bricks; and 6: moving the head performing movements of assent), then we measured the length of the sequence of actions reproduced by the child. Summing the imitated sequences of the three trials, we obtained the *total imitated sequences* to be correlated with neuroanatomical values.

Furthermore, we summed the *manipulations* of both tasks, that is, the total number of object-linked actions not aimed to build the house (e.g., handling the bricks without assembling them, changing their position on the table, and holding the bricks). In other words, we considered as manipulations all actions that – although were not closely related to the specific proposed task (that is to build the house) – can be anyhow considered “play” actions and not “bizarre” actions.

Finally, we summed the *task-irrelevant actions* of both tasks, that is, the total number of weird actions not at all related to the task (e.g., beating the bricks on the table and throwing the bricks to the ground). In other words, we considered as task-irrelevant actions all actions that cannot be considered “play” actions and that clearly broke the experimental setting.

For all parameters, two coders independently attributed the scores. The scoring was considered reliable only when by comparing measures through Cohen’s kappa coefficient their judgments were consistent (*k* > 0.75).

### Images Acquisition

All participants underwent an MRI scan at Bambino Gesù Children’s Hospital, in a window time of 2 weeks from learning tasks. Brain MRI scans were performed at 1.5 T (Siemens, Magnetom Vision, Erlangen, Germany). In a single session, a 3D T1-weighted turbo-flash magnetization prepared rapid-acquisition gradient echo (3D MPRAGE) (TR/TE = 11.4/4.4 ms, TI = 300 ms, flip angle = 158) sequence was obtained from all participants with an isotropic 1 mm^3^ voxel size. Scans were processed at the IRCCS Fondazione Santa Lucia (Rome, Italy).

### Cerebral Measures

Cerebral measures (cortical thickness and gray matter volume) were estimated using FreeSurfer software (FreeSurfer 5.1.0^1^), a widely documented and automated program for the analysis of brain structure. The technical details have been described in previous publications (Dale and Sereno, 1993; Dale et al., 1999; Fischl et al., 1999, 2002, 2004; Fischl and Dale, 2000; Ségonne et al., 2004; Jovicich et al., 2006). Briefly, local cortical thickness was measured by estimating the shortest distance between the position of spatially equivalent surface points on the pial surface and the gray-white matter boundary and vice versa, and averaging the two values (Fischl and Dale, 2000). A 10-mm full-width half-maximum Gaussian kernel was then applied to smooth data.

Hemispheric cortical thickness parcellation on gyral based Desikan-Killiany cortical Atlas is automatically generated by FreeSurfer software. The FreeSurfer implements a technique for automatically assigning a neuroanatomical label to each location on a cortical surface model based on probabilistic information estimated from a manually labeled training set. This procedure incorporates both geometric information derived from the cortical model and neuroanatomical convention, as found in the training set. These are the individual Desikan-Killiany ROI mapped for each lobe: frontal lobe (superior frontal, rostral and caudal middle frontal, pars opercularis, pars triangularis and pars orbitalis, lateral and medial orbitofrontal, precentral, paracentral, and frontal pole); parietal lobe (superior parietal, inferior parietal, supramarginal, postcentral, and precuneus); temporal lobe (superior, middle, and inferior temporal, banks of the superior temporal sulcus, fusiform, transverse temporal, entorhinal, temporal pole, and parahippocampal); occipital lobe (lateral occipital, lingual, cuneus, and pericalcarine) and cingulate if it needed to be included in a lobe (rostral anterior, caudal anterior, posterior, isthmus).

We also obtained (as additional FreeSurfer output) gray matter volume of the following brain regions: nucleus accumbens, caudate nucleus, pallidum, putamen, hippocampus, amygdala, thalamus and cerebellum, together with total intracranial volume.

FreeSurfer outputs were visually inspected and manually edited (when required) by a trained researcher (FP). In order to account for individual variability in head size, local cortical thickness measures were corrected for total hemispheric thickness, while volumetric measures were corrected for total intracranial volume.

According to our *a priori* hypothesis, for the OBS task we selected the following ROI: frontal lobe (pars opercularis, pars triangularis, premotor area), parietal lobe (inferior parietal gyrus), temporal lobe (superior temporal gyrus), and cerebellar hemispheres (Figure 2A). For the EXP task we selected the following ROI: precentral frontal gyrus and superior frontal gyrus, cerebellar hemispheres, basal ganglia (caudate, pallidum, putamen), and thalamus (Figure 2B).

### Statistical Analysis

Behavioral data were first tested for normality (Shapiro–Wilk’s test) and homoscedasticity (Levene’s test) and then compared by using parametric one-way analyses of variance (ANOVAs) with repeated measures, or non-parametric Friedman’s ANOVA, or Mann-Whitney *U* test. Correlations between data were tested by means of Pearson’s r or Spearman’s r. Associations between behavioral parameters and cerebral measures were tested by using stepwise multiple regression analyses (F-to-enter = 1) with each behavioral parameter as dependent variable and volumes and thickness measures as independent variables. Statistical analyses were performed by using Statistica 8.0 for Windows and the significance level was set at *p* < 0.05.

## Results

### Behavioral Results

#### OBS Task

**FIGURE 3:**
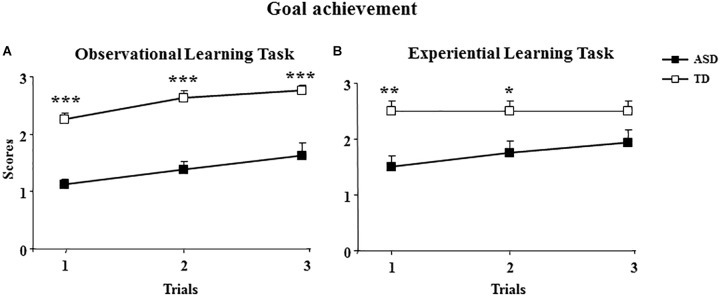
Behavioral results of ASD and TD children. Performance of ASD and TD children in the observational learning task **(A)** and experiential learning task **(B)**. Data are presented as mean ± SEM. Asterisks indicate the significance level of comparisons between groups: ^∗^*p* < 0.05, ^∗∗^*p* < 0.005, and ^∗∗∗^*p* < 0.0005.

Although the performances of the both groups of participants significantly improved in the three trials of the OBS task (Friedman’s ANOVA on goal achievement scores: ASD group, *χ*^2^(2) = 7.11; *p* = 0.03; TD group, *χ*^2^(2) = 13; *p* = 0.001), the children with ASD obtained significantly lower scores on goal achievement parameter than TD children (Mann-Whitney *U* test: 1st trial, *z* = -4.79, *p* = 0.000002; 2nd trial, *z* = -4.19, *p* = 0.00003; 3rd trial: *z* = -3.46, *p* = 0.0005) (Figure 3A).

Interestingly, TD children did not show hyperimitations and imitated sequences as well as manipulations and task-irrelevant actions. So, statistical comparisons between groups on these behavioral parameters were not performed.

In the ASD group, the number of hyperimitations did not significantly change during the three OBS trials [ANOVA: *F*(2, 30) = 1.10; *p* = 0.34], while the number of imitated sequences significantly increased [ANOVA: *F*(2, 30) = 3.64; *p* = 0.04].

**FIGURE 4:**
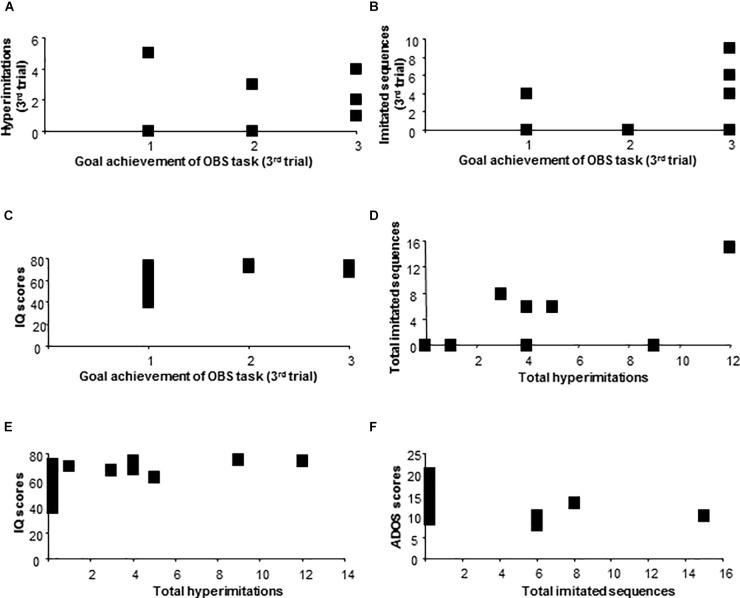
Behavioral results of ASD children. Correlation between goal achievement of OBS task and hyperimitations **(A)**, imitated sequences **(B)**, IQ scores **(C)**. Correlation between total hyperimitations and total imitated sequences **(D)**, IQ scores **(E)**. Correlation between total imitated sequences and ADOS scores **(F)**.

As for the ASD group, at the third trial, goal achievement scores positively correlated with the hyperimitations (Spearman’s *r* = 0.66, *p* = 0.005), with the imitated sequences (Spearman’s *r* = 0.58, *p* = 0.02), with IQ (Spearman’s *r* = 0.56, *p* = 0.02), and did not correlate with total ADOS scores (Spearman’s *r*: -0.22; *p* = 0.39) (Figures 4A–C).

Total hyperimitations of children with ASD positively correlated with total imitated sequences (Pearson’s *r*: 0.73; *p* = 0.001), with IQ (Pearson’s *r* = 0.57; *p* = 0.021) and did not correlate with total ADOS scores (Pearson’s *r*: -0.43; *p* = 0.09). Total imitated sequences of children with ASD negatively correlated with total ADOS scores (Pearson’s *r*: -0.54; *p* = 0.03) and did not correlate with IQ scores (Pearson’s *r*: 0.35; *p* = 0.18) (Figures 4D–F).

#### EXP Task

Children with ASD obtained significantly lower scores of goal achievement than TD children in the first and second trial, but not in the third trial (Mann-Whitney *U* test: 1st trial, *z* = -3.08, *p* = 0.002; 2nd trial, *z* = -2.42, *p* = 0.01; 3rd trial: *z* = -1.76, *p* = 0.08) (Figure 3B).

The performances of the children with ASD significantly improved in the three trials of the EXP task (Friedman’s ANOVA on goal achievement scores: *χ*^2^(2) = 6.61; *p* = 0.03). The goal achievement scores obtained in the last trial by children with ASD did not correlate with IQ (Spearman’s *r* = 0.33, *p* = 0.21) and total ADOS scores (Spearman’s *r*: -0.28; *p* = 0.29).

Goal achievement scores of EXP task positively correlated with the scores obtained in OBS task in the second and third trial in the ASD group, and in the third trial in TD group (ASD group: 1st trial, Spearman’s *r* = 0.20, *p* = 0.46; 2nd trial, *r* = 0.65, *p* = 0.007; 3rd trial, *r* = 0.67, *p* = 0.004; TD group: 1st trial, *r* = 0.44, *p* = 0.09; 2nd trial, *r* = 0.42, *p* = 0.1; 3rd trial, *r* = 0.69, *p* = 0.003).

Furthermore, while the total manipulations of the children with ASD did not significantly change over trials [ANOVA: *F*(2, 30) = 0.14; *p* = 0.86], their total task-irrelevant actions significantly diminished over trials [*F*(2, 30) = 3.63; *p* = 0.03].

### Neuroimaging Results for the ASD Group

#### OBS Task

Associations between behavioral scores of children with ASD in the OBS task and cortical thickness or subcortical regional volumes are reported in Table 2A. Stepwise multiple regression analyses highlighted the significant positive association between goal achievement scores and thickness of the left pars opercularis [beta = 0.87; *t*(11) = 3.66; *p* = 0.0037].

**Table 2:** Associations between behavioral scoresof children with ASD and cortical thickness or subcortical regional volumes.

A. Observational Learning Task			
**Goal achievement**			
Adjusted *R*^2^ = 0.49; *F*(4, 11) = 4.67; *p* = 0.019			

	**Beta**	***t*(11)**	***p***

Left pars opercularis	0.86	3.66	**0.004**
Left cerebellum	-0.30	-1.44	0.18
Left superior temporal cortex	-0.45	-1.82	0.09
Left pars triangularis	-0.31	-1.62	0.13
**Total hyperimitations**			
Adjusted *R*^2^ = 0.52; *F*(3, 12) = 6.5; *p* = 0.007			

	**Beta**	***t*(12)**	***p***

Right superior temporal cortex	0.62	2.79	**0.02**
Left cerebellum	-1.29	-3.19	**0.008**
Right cerebellum	1	2.33	**0.04**
**Total imitated sequences**			
Adjusted *R*^2^ = 0.79; *F*(6, 9) = 10.49; *p* = 0.001			

	**Beta**	***t*(9)**	***p***

Right superior temporal cortex	0.83	4.49	**0.001**
Left cerebellum	-1.36	-4.38	**0.002**
Right cerebellum	0.89	2.36	**0.04**
Left premotor cortex	-0.38	-2.65	**0.03**
Left pars opercularis	0.32	1.8	0.10
Left superior temporal cortex	-0.23	-1.13	0.23

**B. Experiential Learning Task**			

**Goal achievement**			
Adjusted *R*^2^ = 0.86; *F*(9, 6) = 11.07; *p* = 0.004			

	**Beta**	***t*(6)**	***p***

Right thalamus	0.14	0.45	0.67
Left cerebellum	-1.51	-3.67	**0.01**
Right precentral cortex	0.71	2.84	**0.03**
Left precentral cortex	-0.23	-1.33	0.23
Left thalamus	-0.81	-2.72	**0.04**
Left superior frontal cortex	-0.71	-3.78	**0.009**
Left caudate	-0.05	-0.35	0.74
Right cerebellum	1.19	2.43	0.05
Left pallidum	-0.34	-1.88	0.11


*Bold values are statistically significant.*

Significant positive associations emerged also between total hyperimitations and thickness of right superior temporal cortex [beta = 0.622; *t*(12) = 2.79; *p* = 0.016], and gray matter volume of the right cerebellum [beta = 1; *t*(12) = 2.33; *p* = 0.04]. Conversely, total hyperimitations were negatively associated with gray matter volume of the left cerebellum [beta = -1.2; *t*(12) = -3.2; *p* = 0.008].

Total imitated sequences were associated negatively with the volume of left cerebellum [beta = -1.36; *t*(9) = -4.38; *p* = 0.002] and with the thickness of left premotor cortex [beta = -0.38; *t*(9) = -2.65; *p* = 0.026], and positively with gray matter volume of right cerebellum [beta = 0.89; *t*(9) = 2.36; *p* = 0.04] and with the thickness of right superior temporal cortex [beta = 0.83; *t*(9) = 4.5; *p* = 0.002].

#### EXP Task

Associations between behavioral scores of children with ASD in the EXP task and cortical thickness or subcortical regional volumes are reported in Table 2B. Stepwise multiple regression analyses highlighted that goal achievement scores were associated positively with thickness of right precentral cortex [beta = 0.71; *t*(6) = 2.84; *p* = 0.03] and negatively with the thickness of left superior frontal cortex [beta = -0.71; *t*(6) = -3.78; *p* = 0.01], the gray matter volume of the left cerebellum [beta = -1.51; *t*(6) = -3.67; *p* = 0.01] and left thalamus [beta = -0.81; *t*(6) = -2.72; *p* = 0.03].

Moreover, to verify if the correlations were region-specific rather than a mere correlation between behavior and cortical thickness, we correlated the ROI selected for the OBS task resulting significant in the stepwise multiple regression analysis (left pars opercularis, Table 2A) with the goal achievement scores obtained in the EXP task. Vice versa, we correlated the ROI selected for the EXP task resulting significant in the stepwise multiple regression analysis (left cerebellum, right precentral cortex, left thalamus, left superior frontal cortex, Table 2B) with the goal achievement scores obtained in the OBS task. These analyses did not document any significant correlation between the brain region selected for the OBS task and the goal achievement scores of the EXP task (left pars opercularis: Spearman’s *r* = 0.071, *p* = 0.79). Similarly, no correlation between ROI selected for the EXP task and the goal achievement scores of the OBS task was found (left cerebellum: *r* = -0.11, *p* = 0.68; right precentral cortex: *r* = 0.3, *p* = 0.26; left thalamus: *r* = -0.42, *p* = 0.11; left superior frontal cortex: *r* = 0.29, *p* = 0.28).

## Discussion

The current study was aimed at comparing the performances of low-functioning children with ASD with those of TD children in a task of learning by observation and in a task of learning by doing. Moreover, the present study was aimed at correlating the performances of the children with ASD with morphological features of cortical and subcortical regions retained to be involved in such kinds of learning. Currently, there are relatively few neuroanatomical studies examining the brain of low-functioning young children with ASD (Toal et al., 2010; Goldman et al., 2013), and no studies included correlations with their own behavioral performances.

The main result of the present research was that the children with ASD were severely impaired in both OBS and EXP tasks even if their performances improved in the three trials of both tasks. Such an improvement indicates that the construction play represented a challenge with a difficulty level fitting their level of skill. Similarly to TD children, the children with ASD required the actual practice to refine the cognitive representation developed through observation, because response-produced sensory feedback engages the learner in processes not employed during observation (Shea et al., 2000; Weeks and Anderson, 2000; Trempe et al., 2011).

The OBS task required participants to observe a “novice” actor who learned the task committing errors and trying different solutions. This multifaceted observational training was supposed to help the observer children detect the errors and associate behavioral patterns with outcomes (success or failure). Goal achievement scores of the low-functioning children with ASD positively correlated with IQ scores and hyperimitations that in turn positively correlated with IQ scores. Thus, the children with ASD tended to have a “copy-all” approach that reproduced the correct, incorrect and useless actions of the actor. The positive correlations of hyperimitations with IQ and goal achievement scores seem to suggest that in ASD the hyperimitation might represent a functional learning strategy (Hopper et al., 2010; Whiten and Flynn, 2010). Furthermore, our findings indicate that the responses of the low-functioning children with ASD were not driven by the saliency of the actions observed, and that the observation of the actor’s behavior did not guide the children with ASD in determining which behavior was more congruent with the final goal, given they included even the failed attempts of the actor in their response. We found the same propensity to the hyperimitation in a group of high-functioning children with ASD who when learning a visuo-motor sequence by observation also reproduced the patently wrong actor’s actions (Foti et al., 2014). Thus, copying exactly the actor’s actions appears to be a learning strategy employed by both high- and low-functioning children with ASD (Somogyi et al., 2013). Rather than analyzing the context of the observed behavior and selecting the most efficient observed actions to achieve the goal, children with ASD reproduce the entirety of the observed actor’s actions. As pointed out by Gergely et al. (2002), without the ability to take into account the context of the observed behaviors, most social situations become cognitively opaque or difficult to interpret. The strategy of children with ASD seems appropriate for addressing this difficulty. That is, reproducing the entire sequence of observed behavior is a redundant but safe method of producing relevant and efficient actions (Gergely and Csibra, 2006). Interestingly, the inclination to hyperimitate documented in the children with ASD was totally absent in the TD children. Thus, we can advance that such a tendency reflects a syndrome-specific learning strategy not exhibited by the TD children.

Such a propensity to hyperimitation may rely on recruiting brain regions associated with action observation and execution and providing the observer with a matching motor representation in their own motor system. These regions represent the potential MNS in analogy to the mirror neurons found in similar brain regions in monkeys (Rizzolatti et al., 2001; Rizzolatti and Craighero, 2004). Given action mirroring is assumed to underlie a wide range of functions, including imitation and action understanding (Rizzolatti and Craighero, 2004; Fogassi et al., 2005; Iacoboni, 2009), the imitative deficits of children with ASD have been related to abnormalities of their MNS (Williams et al., 2001; Iacoboni and Dapretto, 2006). The present findings are consistent with this idea. In fact, in our group of children with ASD the marked hyperimitative tendencies were positively associated with the thickness of the specific areas belonging to MNS (left pars opercularis, left premotor cortex, and right superior temporal cortex) and with the volume of both cerebellar hemispheres.

Moreover, the significant association between goal achievement scores and thickness of pars opercularis of the inferior frontal gyrus nicely fits with the role attributed to this region in aspects of motor processing, including motor sequence learning, action observation, motor imagery and imitation (Rizzolatti et al., 1996; Grafton et al., 2002; Grèzes and Decety, 2002; Koski et al., 2002; Caspers et al., 2010). Similarly, the specific association between imitated sequences and thickness of premotor cortex is consistent with the role of premotor cortex in sensorimotor transformation, control of goal-directed attention, and instrumental learning (Corbetta and Shulman, 2002; Suzuki and Brown, 2005; Brovelli et al., 2008). The association between hyperimitations and imitated sequences, and thickness of superior temporal sulcus (STS) is consistent with STS role in actively monitoring higher-order visuo-motor correspondences between own actions and actions of others (Iacoboni et al., 2001; Molenberghs et al., 2010). Finally, the specific association between hyperimitations and imitated sequences and the volumes of cerebellar hemispheres is consistent with the role of cerebellar hemispheres (namely, Crus I and II regions) in observation and imitative execution of biological movements (Kessler et al., 2006). Interestingly, while healthy subjects exhibit increased connectivity between cerebellar hemispheres and STS during imitation, in individuals with ASD a disconnectivity between cerebellar hemispheres and multiple cortical areas involved in imitation has been described (Mostofsky et al., 2006). This alteration has been hypothesized to be at the base of impaired profile of imitation in ASD (Dziuk et al., 2007; Hagura et al., 2009).

It is noteworthy that since any learning by observation requires attending to and encoding the relevant actions to be imitated, the altered attention to social stimuli [e.g., “weak central coherence” (Happé and Frith, 2006) and “enhanced perceptual functioning” (Mottron et al., 2006)] described in children with ASD could interfere with their observational learning. Notably, cortical areas associated with the MNS heavily overlap with the brain areas associated with attentional reorienting. Namely, the inferior frontal gyrus is involved in attentional reorienting and in inhibiting irrelevant information during imitation, the STS is involved in processing eye gaze and in inferring direction of attention of the other person (Materna et al., 2008), and also the cerebellum has been involved in drawing attentional resources (Akshoomoff et al., 1997; Townsend et al., 1999) and thus it may contribute to attentional problems in autism (Allen and Courchesne, 2001; Townsend et al., 2001).

In contrast (or in addition) to the hypothesis that the atypical observational learning of children with ASD may rely on atypical MNS and other associated regions, another hypothesis suggests that autistic individuals should have problems in the control of imitative behavior and in the distinction between the self and other agents rather than in imitation *per se* (Spengler et al., 2010). Following this interpretation, in ASD the control over MNS output or top-down modulation of this system could be abnormal (Hamilton et al., 2007; Hamilton, 2008; Kana et al., 2011; Cook et al., 2012, 2014). Thus, children with ASD could have deficits in imitation inhibition and difficulty applying top-down selection. The present behavioral results leave open this possible interpretation, showing that the imitative strategy of children with ASD consisted of reproducing the full set of actions observed, without any selectivity.

As a final note, the differences in cortical thickness and subcortical volumes found in the present research might be due to primary developmental histo-pathological abnormalities (in neuronal proliferation or migration, cell density, cortical microcolumns) alternatively or in combination to secondary consequences of abnormal input to specific brain areas. Consistently with the present results, a very recent study (Yang et al., 2016) described in high-functioning children with ASD abnormal expanded in cortical thickness in posterior superior temporal sulcus and middle temporal gyrus. Recent researches (Zielinski et al., 2014; Wallace et al., 2015) report an increase in age-related cortical thinning in ASD during adolescence. Combining these findings with ours, it seems reasonable to propose that the cortical thickening (or the lack of age-related cortical thinning) in ASD during childhood [our findings and by Yang et al. (2016)] does not continue indefinitely and it is probably followed by a rebound effect of increased cortical thinning during adolescence (Zielinski et al., 2014; Wallace et al., 2015).

It should be remembered, however, that a decrease or an increase in size (thickness or volume) in a given structure does not necessarily mean a decrease or an increase in activity of that region. Cortical thickening could be associated with higher resting baseline or alternatively with decreased activity, if it is due to more inhibitory interneurons or dendritic pruning. To characterize the relationship between structure and function in relation to ASD, it is necessary to distinguish among the different structural gray matter compartments (neurons, interneurons, glia, and neuropil) that contribute to the thickness and volume of the brain regions. Identifying the micro-structural features of specific brain regions could shed light on abnormalities in cognitive, emotional and social processing characterizing ASD.

## Conclusion

We found that performances of the low-functioning children with ASD improved in both OBS and EXP tasks, as trials went by. In the OBS task, the children with ASD tended to have a “copy-all” approach that facilitated the goal achievement. The marked hyperimitative tendencies of the children with ASD were positively associated with the thickness of left pars opercularis, left premotor cortex, and right superior temporal cortex, areas belonging to mirror neuron system, and with the volume of both cerebellar hemispheres. Overall, these findings suggest that in children with ASD the hyperimitation might represent an actual learning strategy.

Finally, since observational learning is pervasive in daily life and crucial for developing complex abilities, clarifying the observational learning deficits in ASD may have several implications for educational and clinical interventions aimed at improving daily functioning in children at risk, as children with ASD may be. Specifically, the present outcomes can be used to develop optimal educational interventions tailored to each child in order to facilitate the acquisition of new cognitive and motor competencies, grant better social integration, and enhance self-efficacy and self-confidence. Moreover, the deep characterization on how children with ASD learn from others may not only allow a best teaching approach, but also provide advances within the development of video-assisted learning reducing the burden for their caregivers.

## Author Contributions

All authors designed the research and wrote and approved the manuscript. FF, LM, and DM gathered the behavioral data. FF, FP, and DM analyzed behavioral and neuroimaging data.

## Conflict of Interest Statement

The authors declare that the research was conducted in the absence of any commercial or financial relationships that could be construed as a potential conflict of interest. The reviewer FF declared a shared affiliation, with no collaboration, with one of the authors, LP, to the handling Editor at the time of review.

## Footnotes

1 http://surfer.nmr.mgh.harvard.edu/

## References

[B1] Akshoomoff N. A. Courchesne E. Townsend J. (1997). Attention coordination and anticipatory control. *Int. Rev. Neurobiol.* 41 575–598. 10.1016/S0074-7742(08)60371-2 9378609

[B2] Allen G. Courchesne E. (2001). Attention function and dysfunction in autism. *Front. Biosci.* 6:D105–D119. 10.2741/allen 11171544

[B3] American Psychiatric Association [APA] (2000). *Diagnostic and Statistical Manual of Mental Disorders* 4th Edn. Washington, DC: American Psychiatric Association.

[B4] Ayres K. M. Langone J. (2005). Intervention and instruction with video for students with autism: a review of the literature. *Educ. Train. Dev. Disabil.* 40 183–196.

[B5] Bandura A. (1977). *Social Learning Theory.* Englewood Cliffs, NJ: Prentice Hall.

[B6] Bellini S. Akullian J. (2007). A meta-analysis of video modelling and video self-modelling interventions for children and adolescents with autism spectrum disorders. *Except. Child.* 73 264–287. 10.1177/001440290707300301

[B7] Brass M. Heyes C. (2005). Imitation: is cognitive neuroscience solving the correspondence problem? *Trends Cogn. Sci.* 9 489–495. 16126449 10.1016/j.tics.2005.08.007

[B8] Brovelli A. Laksiri N. Nazarian B. Meunier M. Boussaoud D. (2008). Understanding the neural computations of arbitrary visuomotor learning through fMRI and associative learning theory. *Cereb. Cortex* 18 1485–1495. 10.1093/cercor/bhm198 18033767

[B9] Buchanan J. J. Dean N. J. (2010). Specificity in practice benefits learning in novice models and variability in demonstration benefits observational practice. *Psychol. Res.* 74 313–326. 10.1007/s00426-009-0254-y 19727806

[B10] Bunge S. A. (2004). How we use rules to select actions: a review of evidence from cognitive neuroscience. *Cogn. Affect. Behav. Neurosci.* 4 564–579. 10.3758/CABN.4.4.564 15849898

[B11] Bunge S. A. Kahn I. Wallis J. D. Miller E. K. Wagner A. D. (2003). Neural circuits subserving the retrieval and maintenance of abstract rules. *J. Neurophysiol.* 90 3419–3428. 10.1152/jn.00910.2002 12867532

[B12] Calderoni S. Retico A. Biagi L. Tancredi R. Muratori F. Tosetti M. (2012). Female children with autism spectrum disorder: an insight from mass-univariate and pattern classification analyses. *Neuroimage* 59 1013–1022. 10.1016/j.neuroimage.2011.08.070 21896334

[B13] Caspers S. Zilles K. Laird A. R. Eickhoff S. B. (2010). ALE meta-analysis of action observation and imitation in the human brain. *Neuroimage* 50 1148–1167. 10.1016/j.neuroimage.2009.12.112 20056149 PMC4981639

[B14] Charlop-Christy M. Le L. Freeman K. (2000). A comparison of video modelling with in vivo modelling for teaching children with autism. *J. Autism Dev. Disord.* 30 537–555. 10.1023/A:1005635326276 11261466

[B15] Cook J. Barbalat G. Blakemore S. J. (2012). Top-down modulation of the perception of other people in schizophrenia and autism. *Front. Hum. Neurosci.* 6:175. 10.3389/fnhum.2012.00175 22715325 PMC3375615

[B16] Cook J. Swapp D. Pan X. Bianchi-Berthouze N. Blakemore S. J. (2014). Atypical interference effect of action observation in autism spectrum conditions. *Psychol. Med.* 44 731–740. 10.1017/S0033291713001335 23759288 PMC3898726

[B17] Corbetta M. Shulman G. L. (2002). Control of goal-directed and stimulus-driven attention in the brain. *Nat. Rev. Neurosci.* 3 201–215. 10.1038/nrn755 11994752

[B18] Crone E. A. Wendelken C. Donohue S. E. Bunge S. A. (2006). Neural evidence for dissociable components of task-switching. *Cereb. Cortex* 16 475–486. 10.1093/cercor/bhi127 16000652

[B19] Dale A. M. Fischl B. Sereno M. I. (1999). Cortical surface-based analysis. I. Segmentation and surface reconstruction. *Neuroimage* 9 179–194. 10.1006/nimg.1998.0395 9931268

[B20] Dale A. M. Sereno M. I. (1993). Improved localizadon of cortical activity by combining EEG and MEG with MRI cortical surface reconstruction: a linear approach. *J. Cogn. Neurosci.* 5 162–176. 10.1162/jocn.1993.5.2.162 23972151

[B21] Dapretto M. Davies M. S. Pfeifer J. H. Scott A. A. Sigman M. Bookheimer S. Y. (2006). Understanding emotions in others: mirror neuron dysfunction in children with autism spectrum disorders. *Nat. Neurosci.* 9 28–30. 10.1038/nn1611 16327784 PMC3713227

[B22] Delano M. E. (2007). Video modelling interventions for individuals with autism. *Remed. Special Educ.* 28 33–42. 10.1177/07419325070280010401

[B23] Donohue S. E. Wendelken C. Crone E. A. Bunge S. A. (2005). Retrieving rules for behavior from long-term memory. *Neuroimage* 26 1140–1149. 10.1016/j.neuroimage.2005.03.019 15961050

[B24] Doyon J. Penhune V. Ungerleider L. G. (2003). Distinct contribution of the cortico-striatal and cortico-cerebellar systems to motor skill learning. *Neuropsychologia* 41 252–262. 10.1016/S0028-3932(02)00158-6 12457751

[B25] Dziuk M. A. Gidley Larson J. C. Apostu A. Mahone E. M. Denckla M. B. Mostofsky S. H. (2007). Dyspraxia in autism: association with motor, social, and communicative deficits. *Dev. Med. Child Neurol.* 49 734–739. 10.1111/j.1469-8749.2007.00734.x 17880641

[B26] Fan Y. T. Decety J. Yang C. Y. Liu J. L. Cheng Y. (2010). Unbroken mirror neurons in autism spectrum conditions. *J. Child Psychol. Psychiatry* 51 981–988. 10.1111/j.1469-7610.2010.02269.x 20524939

[B27] Fischl B. Dale A. M. (2000). Measuring the thickness of the human cerebral cortex from magnetic resonance images. *Proc. Natl. Acad. Sci. U.S.A.* 97 11050–11055. 10.1073/pnas.200033797 10984517 PMC27146

[B28] Fischl B. Salat D. H. Busa E. Albert M. Dieterich M. Haselgrove C. (2002). Whole brain segmentation: automated labeling of neuroanatomical structures in the human brain. *Neuron* 33 341–355. 10.1016/S0896-6273(02)00569-X 11832223

[B29] Fischl B. Sereno M. I. Tootell R. B. Dale A. M. (1999). High-resolution intersubject averaging and a coordinate system for the cortical surface. *Hum. Brain Mapp.* 8 272–284. 10.1002/(SICI)1097-0193(1999)8:4<272::AID-HBM10>3.0.CO;2-4 10619420 PMC6873338

[B30] Fischl B. van der Kouwe A. Destrieux C. Halgren E. Ségonne F. Salat D. H. (2004). Automatically parcellating the human cerebral cortex. *Cereb. Cortex* 14 11–22. 10.1093/cercor/bhg087 14654453

[B31] Fogassi L. Ferrari P. F. Gesierich B. Rozzi S. Chersi F. Rizzolatti G. (2005). Parietal lobe: from action organization to intention understanding. *Science* 308 662–667. 10.1126/science.1106138 15860620

[B32] Foti F. Mazzone L. Menghini D. De Peppo L. Federico F. Postorino V. (2014). Learning by observation in children with autism spectrum disorder. *Psychol. Med.* 44 2437–2447. 10.1017/S003329171300322X 24433947

[B33] Gallese V. Goldman A. (1998). Mirror neurons and the simulation theory of mind-reading. *Trends Cogn. Sci.* 2 493–501. 10.1016/S1364-6613(98)01262-5 21227300

[B34] Gergely G. Bekkering H. Király I. (2002). Rational imitation in preverbal infants. *Nature* 415:755. 10.1038/415755a 11845198

[B35] Gergely G. Csibra G. (2006). “Sylvia’s recipe: the role of imitation and pedagogy in the transmission of human culture,” in *Roots of Human Sociality: Culture, Cognition, and Human Interaction*, eds Enfield N. J. Levinson S. C. (Oxford: Berg Publishers), 229–255.

[B36] Goldman S. O’Brien L. M. Filipek P. A. Rapin I. Herbert M. R. (2013). Herbert motor stereotypies and volumetric brain alterations in children with autistic disorder. *Res. Autism Spectr. Disord.* 7 82–92. 10.1016/j.rasd.2012.07.005 23637709 PMC3639008

[B37] Grafton S. T. Hazeltine E. Ivry R. B. (2002). Motor sequence learning with the nondominant left hand. A PET functional imaging study. *Exp. Brain Res.* 146 369–378. 10.1007/s00221-002-1181-y 12232693

[B38] Grèzes J. Decety J. (2002). Does visual perception of object afford action? Evidence from a neuroimaging study. *Neuropsychologia* 40 212–222. 10.1016/S00283932(01)00089-6 11640943

[B39] Hagura N. Oouchida Y. Aramaki Y. Okada T. Matsumura M. Sadato N. (2009). Visuokinesthetic perception of hand movement is mediated by cerebro-cerebellar interaction between the left cerebellum and right parietal cortex. *Cereb. Cortex* 19 176–186. 10.1093/cercor/bhn068 18453537 PMC2638744

[B40] Hamilton A. F. (2008). Emulation and mimicry for social interaction: a theoretical approach to imitation in autism. *Q. J. Exp. Psychol.* 61 101–115. 10.1080/17470210701508798 18038342

[B41] Hamilton A. F. (2015). Cognitive underpinnings of social interaction. *Q. J. Exp. Psychol.* 68 417–432. 10.1080/17470218.2014.973424 25405540

[B42] Hamilton A. F. Brindley R. M. Frith U. (2007). Imitation and action understanding in autistic spectrum disorders: how valid is the hypothesis of a deficit in the mirror neuron system? *Neuropsychologia* 45 1859–1868. 17234218 10.1016/j.neuropsychologia.2006.11.022

[B43] Happé F. Frith U. (2006). The weak coherence account: detail-focused cognitive style in autism spectrum disorders. *J. Autism Dev. Disord.* 36 5–25. 10.1007/s10803-005-0039-0 16450045

[B44] Hayne H. Herbert J. Simcock G. (2003). Imitation from television by 24- and 30-month-olds. *Dev. Sci.* 6 254–261. 10.1111/1467-7687.00281

[B45] Hazlett H. C. Poe M. D. Gerig G. Styner M. Chappell C. Smith R. G. (2011). Early brain overgrowth in autism associated with an increase in cortical surface area before age 2 years. *Arch. Gen. Psychiatry* 68 467–476. 10.1001/archgenpsychiatry.2011.39 21536976 PMC3315057

[B46] Hopper L. M. Flynn E. G. Wood L. A. Whiten A. (2010). Observational learning of tool use in children: investigating cultural spread through diffusion chains and learning mechanisms through ghost displays. *J. Exp. Child Psychol.* 106 82–97. 10.1016/j.jecp.2009.12.001 20064644

[B47] Iacoboni M. (2005). Neural mechanisms of imitation. *Curr. Opin. Neurobiol.* 15 632–637. 10.1016/j.conb.2005.10.010 16271461

[B48] Iacoboni M. (2009). Neurobiology of imitation. *Curr. Opin. Neurobiol.* 2009 661–665. 10.1016/j.conb.2009.09.008 19896362

[B49] Iacoboni M. Dapretto M. (2006). The mirror neuron system and the consequences of its dysfunction. *Nat. Rev. Neurosci.* 7 942–951. 10.1038/nrn2024 17115076

[B50] Iacoboni M. Koski L. M. Brass M. Bekkering H. Woods R. P. Dubeau M. C. (2001). Reafferent copies of imitated actions in the right superior temporal cortex. *Proc. Natl. Acad. Sci. U.S.A.* 98 13995–13999. 10.1073/pnas.241474598 11717457 PMC61155

[B51] Jovicich J. Czanner S. Greve D. Haley E. van der Kouwe A. Gollub R. (2006). Reliability in multi-site structural MRI studies: effects of gradient non-linearity correction on phantom and human data. *Neuroimage* 30 436–443. 10.1016/j.neuroimage.2005.09.046 16300968

[B52] Kana R. K. Wadsworth H. M. Travers B. G. (2011). A systems level analysis of the mirror neuron hypothesis and imitation impairments in autism spectrum disorders. *Neurosci. Biobehav. Rev.* 35 894–902. 10.1016/j.neubiorev.2010.10.007 20974171

[B53] Kessler K. Biermann-Ruben K. Jonas M. Siebner H. R. Bäumer T. Münchau A. (2006). Investigating the human mirror neuron system by means of cortical synchronization during the imitation of biological movements. *Neuroimage* 33 227–238. 10.1016/j.neuroimage.2006.06.014 16876435

[B54] Klein A. M. Hauf P. Ascherleben G. (2006). The role of action effects in 12-month-olds’ action control: a comparison of televised model and live model. *Infant Behav. Dev.* 29 535–544. 10.1016/j.infbeh.2006.07.001 17138306

[B55] Koski L. Wohlschläger A. Bekkering H. Woods R. P. Dubeau M. C. Mazziotta J. C. (2002). Modulation of motor and premotor activity during imitation of target-directed actions. *Cereb. Cortex* 12 847–855. 10.1093/cercor/12.8.847 12122033

[B56] Ledford J. R. Gast D. L. Luscre D. Ayres K. M. (2008). Observational and incidental learning by children with autism during small group instruction. *J. Autism Dev. Disord.* 38 86–103. 10.1007/s10803-007-0363-7 17347879

[B57] Leighton J. Bird G. Charman T. Heyes C. (2008). Weak imitative performance is not due to a functional “mirroring” deficit in adults with autism spectrum disorders. *Neuropsychologia* 46 1041–1049. 10.1016/j.neuropsychologia.2007.11.013 18177677

[B58] Lord C. Rutter M. Di Lovore P. C. Risi S. (2005). *Autism Diagnostic Observation Schedule.* Florence: Organizzazioni Speciali.

[B59] Luiz D. Barnard A. Knosen N. Kotras N. Horrocks S. McAlinden P. (2006). *GMDS-ER 2-8 Griffith Mental Developmental Scales-Extended Revised: 2 to 8 Years.* Oxford: The Test Agency.

[B60] Materna S. Dicke P. W. Thier P. (2008). Dissociable roles of the superior temporal sulcus and the intraparietal sulcus in joint attention: a functional magnetic resonance imaging study. *J. Cogn. Neurosci.* 20 108–119. 10.1162/jocn.2008.20008 18095789

[B61] McCoy K. Hermansen E. (2007). Video modelling for individuals with autism. a review of model types and effects. *Educ. Treat Child.* 30 183–213. 10.1353/etc.2007.0029

[B62] Meltzoff A. N. Decety J. (2003). What imitation tells us about social cognition: a rapprochement between developmental psychology and cognitive neuroscience. *Philos. Trans. R. Soc. Lond. B Biol. Sci.* 358 491–500. 10.1098/rstb.2002.1261 12689375 PMC1351349

[B63] Meltzoff A. N. Kuhl P. K. Movellan J. Sejnowski T. J. (2009). Foundations for a new science of learning. *Science* 325 284–288. 10.1126/science.1175626 19608908 PMC2776823

[B64] Molenberghs P. Brander C. Mattingley J. B. Cunnington R. (2010). The role of the superior temporal sulcus and the mirror neuron system in imitation. *Hum. Brain Mapp.* 31 1316–1326. 10.1002/hbm.20938 20087840 PMC6870593

[B65] Mostofsky S. H. Dubey P. Jerath V. K. Jansiewicz E. M. Goldberg M. C. Denckla M. B. (2006). Developmental dyspraxia is not limited to imitation in children with autism spectrum disorders. *J. Int. Neuropsychol. Soc.* 12 314–326. 10.1017/S1355617706060437 16903124

[B66] Mottron L. Dawson M. Soulières I. Hubert B. Burack J. (2006). Enhanced perceptual functioning in autism: an update, and eight principles of autistic perception. *J. Autism Dev. Disord.* 36 27–43. 10.1007/s10803-005-0040-7 16453071

[B67] Mundy P. (2011). “The social behavior of autism: a parallel and distributed information processing perspective,” in *Autism Spectrum Disorders* eds Amaral D. G. Dawson G. Geschwind D. H. (New York, NY: Oxford University Press) 149–171.

[B68] Nadel J. (2015). Perception-action coupling and imitation in autism spectrum disorder. *Dev. Med. Child Neurol.* 57 55–58. 10.1111/dmcn.12689 25690119

[B69] Nadel J. Aouka N. Coulon N. Gras-Vincendon A. Canet P. Fagard J. (2011). Yes they can! An approach to observational learning in low-functioning children with autism. *Autism* 15 421–435. 10.1177/1362361310386508 21454387

[B70] Oberman L. M. Hubbard E. M. McCleery J. P. Altschuler E. L. Ramachandran V. S. Pineda J. A. (2005). EEG evidence for mirror neuron dysfunction in autism spectrum disorders. *Brain Res. Cogn. Brain Res.* 24 190–198. 10.1016/j.cogbrainres.2005.01.014 15993757

[B71] Oberman L. M. Ramachandran V. S. Pineda J. A. (2008). Modulation of mu suppression in children with autism spectrum disorders in response to familiar or unfamiliar stimuli: the mirror neuron hypothesis. *Neuropsychologia* 46 1558–1565. 10.1016/j.neuropsychologia.2008.01.010 18304590

[B72] Petrosini L. (2007). ‘Do what I do’ and ‘do how I do’: different components of imitative learning are mediated by different neural structures. *Neuroscientist* 13 335–348. 10.1177/10738584070130040701 17644765

[B73] Plavnick J. B. Hume K. A. (2014). Observational learning by individuals with autism: a review of teaching strategies. *Autism* 18 458–466. 10.1177/1362361312474373 24101717

[B74] Rizzolatti G. Craighero L. (2004). The mirror-neuron system. *Annu. Rev. Neurosci.* 27 169–192. 10.1146/annurev.neuro.27.070203.144230 15217330

[B75] Rizzolatti G. Fadiga L. Gallese V. Fogassi L. (1996). Premotor cortex and the recognition of motor actions. *Brain Res. Cogn. Brain Res.* 3 131–141. 10.1016/0926-6410(95)00038-0 8713554

[B76] Rizzolatti G. Fogassi L. Gallese V. (2001). Neurophysiological mechanisms underlying the understanding and imitation of action. *Nat. Rev. Neurosci.* 2 661–670. 10.1038/35090060 11533734

[B77] Rizzolatti G. Sinigaglia C. (2010). The functional role of the parieto-frontal mirror circuit: interpretations and misinterpretations. *Nat. Rev. Neurosci.* 11 264–274. 10.1038/nrn2805 20216547

[B78] Rogers S. J. Williams J. H. G. (2006). *Imitation and the Social Mind: Autism and Typical Development.* New York, NY: Guilford Press.

[B79] Schumann C. M. Bloss C. S. Barnes C. C. Wideman G. M. Carper R. A. Akshoomoff N. (2010). Longitudinal magnetic resonance imaging study of cortical development through early childhood in autism. *J. Neurosci.* 30 4419–4427. 10.1523/JNEUROSCI.5714-09.2010 20335478 PMC2859218

[B80] Ségonne F. Dale A. M. Busa E. Glessner M. Salat D. Hahn H. K. (2004). A hybrid approach to the skull stripping problem in MRI. *Neuroimage* 22 1060–1075. 10.1016/j.neuroimage.2004.03.032 15219578

[B81] Shea C. H. Wright D. L. Wulf G. Whitacre C. (2000). Physical and observational practice afford unique learning opportunities. *J. Mot. Behav.* 32 27–36. 10.1080/00222890009601357 11008269

[B82] Somogyi E. Király I. Gergely G. Nadel J. (2013). Understanding goals and intentions in low-functioning autism. *Res. Dev. Disabil.* 34 3822–3832. 10.1016/j.ridd.2013.07.039 24021392

[B83] Sparrow S. Balla D. Cicchetti D. (1994). *Vineland Adaptive Behaviour Scale (Survey Form).* Circle Pines, MN: American Guidance Service.

[B84] Spengler S. Bird G. Brass M. (2010). Hyperimitation of actions is related to reduced understanding of others’ minds in autism spectrum conditions. *Biol. Psychiatry* 68 1148–1155. 10.1016/j.biopsych.2010.09.017 21130224

[B85] Suzuki W. Brown E. (2005). Behavioral and neurophysiological analyses of dynamic learning processes. *Behav. Cogn. Neurosci. Rev.* 4 67–95. 10.1177/1534582305280030 16251726

[B86] Taylor B. A. DeQuinzio J. A. (2012). Observational learning and children with autism. *Behav. Modif.* 36 341–360. 10.1177/0145445512443981 22569578

[B87] Toal F. Daly E. M. Page L. Deeley Q. Hallahan B. Bloemen O. (2010). Clinical and anatomical heterogeneity in autistic spectrum disorder: a structural MRI study. *Psychol. Med.* 40 1171–1181. 10.1017/S0033291709991541 19891805

[B88] Townsend J. Courchesne E. Covington J. Westerfield M. Harris N. S. Lyden P. (1999). Spatial attention deficits in patients with acquired or developmental cerebellar abnormality. *J. Neurosci.* 19 5632–5643. 10.1523/JNEUROSCI.19-13-05632.1999 10377369 PMC6782343

[B89] Townsend J. Westerfield M. Leaver E. Makeig S. Jung T. Pierce K. (2001). Event-related brain response abnormalities in autism: evidence for impaired cerebello-frontal spatial attention networks. *Brain Res. Cogn. Brain Res.* 11 127–145. 10.1016/S0926-6410(00)00072-0 11240116

[B90] Trempe M. Sabourin M. Rohbanfard H. Proteau L. (2011). Observation learning versus physical practice leads to different consolidation outcomes in a movement timing task. *Exp. Brain Res.* 209 181–192. 10.1007/s00221-011-2540-3 21279634

[B91] Vivanti G. Dissanayake C. (2014). Propensity to imitate in autism is not modulated by the model’s gaze direction: an eye-tracking study. *Autism Res.* 7 392–399. 10.1002/aur.1376 24740914

[B92] Vivanti G. Hocking D. R. Fanning P. Dissanayake C. (2016). Social affiliation motives modulate spontaneous learning in Williams syndrome but not in autism. *Mol. Autism* 7:40. 10.1186/s13229-016-0101-0 27610215 PMC5015226

[B93] Vivanti G. Trembath D. Dissanayake C. (2014). Mechanisms of imitation impairment in autism spectrum disorder. *J. Abnorm. Child Psychol.* 42 1395–1405. 10.1007/s10802-014-9874-9 24736983

[B94] Wallace G. L. Eisenberg I. W. Robustelli B. Dankner N. Kenworthy L. Giedd J. N. (2015). Longitudinal cortical development during adolescence and young adulthood in autism spectrum disorder: increased cortical thinning but comparable surface area changes. *J. Am. Acad. Child Adolesc. Psychiatry* 54 464–469. 10.1016/j.jaac.2015.03.007 26004661 PMC4540060

[B95] Weeks D. L. Anderson L. P. (2000). The interaction of observational learning with overt practice: effects on motor skill learning. *Acta Psychol.* 104 259–271. 10.1016/S0001-6918(00)00039-1 10900708

[B96] Whiten A. Flynn E. (2010). The transmission and evolution of experimental microcultures in groups of young children. *Dev. Psychol.* 46 1694–1709. 10.1037/a0020786 20822212

[B97] Williams J. H. Whiten A. Singh T. (2004). A systematic review of action imitation in autistic spectrum disorder. *J. Autism. Dev. Disord.* 34 285–299. 10.1023/B:JADD.0000029551.56735.3a 15264497

[B98] Williams J. H. Whiten A. Suddendorf T. Perrett D. I. (2001). Imitation, mirror neurons and autism. *Neurosci. Biobehav. Rev.* 25 287–295. 10.1016/S0149-7634(01)00014-8 11445135

[B99] Yang D. Y. Beam D. Pelphrey K. A. Abdullahi S. Jou R. J. (2016). Cortical morphological markers in children with autism: a structural magnetic resonance imaging study of thickness, area, volume, and gyrification. *Mol. Autism* 7:11. 10.1186/s13229-016-0076-x 26816612 PMC4727390

[B100] Zielinski B. A. Prigge M. B. Nielsen J. A. Froehlich A. L. Abildskov T. J. Anderson J. S. (2014). Longitudinal changes in cortical thickness in autism and typical development. *Brain* 137 1799–1812. 10.1093/brain/awu083 24755274 PMC4032101

